# An empirical measure of nonlinear strain for soft tissue indentation

**DOI:** 10.1098/rsos.170894

**Published:** 2017-11-01

**Authors:** D. B. MacManus, M. D. Gilchrist, J. G. Murphy

**Affiliations:** 1 Department of Mechanical and Materials Engineering, University College Dublin, Belfield, Dublin 4, Ireland; 2 Department of Mechanical Engineering, Dublin City University, Glasnevin, Dublin 9, Ireland

**Keywords:** brain tissue, strain measures, finite-element simulations

## Abstract

Indentation is a primary tool in the investigation of the mechanical properties of very soft tissue such as the brain. However, the usual material characterization protocols are not applicable because the resulting deformation is inhomogeneous, with even the identification of the amount of strain ambiguous and uncertain. Focusing on spherical indentation only, a standard is needed to quantify the amount of strain in terms of the probe radius and displacement so that different indentation experiments can be compared and contrasted. It is shown here that the minimum axial value of the Eulerian logarithmic strain tensor has many desirable properties of such a standard, such as invariance under the choice of material model, and experimental conditions for a given probe displacement. The disadvantage of this measure is that sophisticated finite element techniques need to be used in its determination. An empirical relation is obtained between this strain and the probe radius and displacement to circumvent this problem, and it is shown that this relationship is an excellent predictor of the strain measure. Two essential features of this empirical measure for nonlinear strains are that the exact strain measure for the linear theory is recovered on restriction to infinitesimal deformations and that the simulations use models based on reliable and accurate indentation data obtained from freshly harvested murine brains using a bespoke micro-indentation device.

## 1. Introduction

It is extremely difficult to measure the mechanical response of brain tissue using the usual material characterization tests such as simple tension and shear. This is because its exceptionally high water content makes it difficult to handle, difficult to cut into regular shapes for testing purposes and difficult to bind to an experimental apparatus, especially with extremely hydrophilic surgical glue. Conversely, indentation experiments are relatively easy to perform, with no need now to bind the material specimen to an experimental apparatus. Another advantage of indentation testing is that its resolution can be increased by simply decreasing the size of the probe. This potentially allows for material constants to be estimated from much smaller specimens than those typically used in the standard tests such as simple and biaxial tension and simple shear. Additionally, micro-indentation has the ability to characterize highly localized mechanical responses of larger specimens. These advantages have led to the wide adoption of indentation techniques to quantify the mechanical response of very soft biological tissue, such as the brain. Some important examples include [1–7].

**Figure 1.**
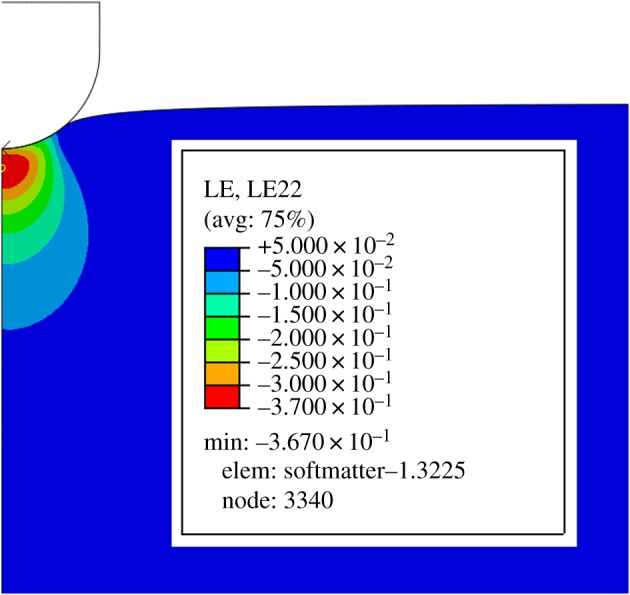
Finite element simulation of a neo-Hookean half-space by a spherical indenter. Indentation results in a complex, inhomogeneous strain field and inverse finite element methods are usually employed to estimate material constants. The strain field plotted here is the normal component in the direction of the applied force of the logarithmic strain tensor, which is discussed in §2.

A major disadvantage of indentation testing is that identifying material constants from the acquired data can be a formidable technical challenge. Indentation results in a complex, inhomogeneous strain field, with the result that extracting material constants from the force–displacement data usually recorded for indentation experiments can be difficult, even for probes with a simple geometry such as the spherical probes considered here. The scale of the inhomogeneity can be judged from figure 1. This means that the constitutive modelling protocols that are valid for homogeneous deformations characteristic of simple tension and shear, for example, cannot be applied to indentation data and, typically, bespoke inverse techniques using finite elements must be employed.

A more fundamental problem with using indentation data to characterize the mechanical response of soft tissue is that the basic concept of strain is not immediately quantifiable. Strain can be defined in a solid body as the ratio of a typical displacement of the body and a natural length-scale of the undeformed body. This typical displacement can be identified with the displacement of the probe *D*, which is one of the measured quantities in indentation experiments. However, there is no natural length-scale for the specimen being tested if it is idealized as being a half-space, as is usually the case. Perhaps the most reliable current method is a recourse to finite element simulations, but this is doubly problematic. It requires access to commercial finite element software and a sophisticated skill set because reverse techniques need to be employed. The second problem is that, even if the first set of problems can be overcome, there is an infinity of strain measures that could be used as a result of the nonlinearity of the experiment and the inhomogeneity of the deformation.

Having a robust measure of strain is important in material characterization studies for several reasons as follows:

1. to normalize the amount of deformation and allow for comparison with other studies in the literature;
2. to calculate the applied strain rate;
3. to verify if indentation experiments are being performed under infinitesimal or finite strains;
4. soft biological tissues typically exhibit rate-dependent properties.

There is therefore a crucial need to establish a robust measure of strain that is readily available to experimentalists performing indentation experiments, ideally one that can be defined in terms of the probe displacement *D* and the radius of the probe *R*, and that will serve as a standard measure of strain for indentation experiments. A number of physically realistic assumptions will be made in order to develop such a measure. The first is that brain tissue is incompressible, motivated by its high water content, with this idealization weakened to slight compressibility in the finite element simulations to overcome the numerical difficulties associated with enforcing incompressibility locally. The second is to assume that brain tissue is isotropic. Although at first glance this might not seem to be as realistic as the incompressibility assumption, it is widely accepted that grey matter is essentially an isotropic material [8]. The degree of anisotropy characteristic of aggregate brain tissue is still moot, as can be seen, for example, in Prange & Margulies [9] and Budday *et al.* [8]. Furthermore, Samadi-Dooki *et al.* [1] found grey matter to exhibit some degree of anisotropy. However, our own experimental findings suggest that isotropy is, in fact, consistent with the mechanical response of brain tissue and, whatever the final consensus on this question, isotropy would appear to be an excellent zero-order approximation to its material symmetry.

A standard measure of indentation strain for very soft tissue should have the following properties:

1. it should be immediately accessible and easily calculated in a laboratory setting;
2. it should have the mathematical properties of a norm;
3. it should be an output from simulations using commercial finite element packages;
4. it should have an intuitive appeal as being representative of the strain field in indentation experiments;
5. it should be robust.

Robustness is defined here as being insensitive to small changes in the protocol of obtaining the standard measure. In particular, the standard measure should have the following sub-properties:

1. when the strain measure is plotted against probe displacement, the strain is relatively invariant under changes in the probe radius;
2. if different material models are fitted to the same force–displacement experimental data, the strain is again invariant under the choice of material when plotted against *D*;
3. the strain measure is invariant under change in the value of Poisson’s ratio, close to $\frac{1}{2}$, recalling that the specimen has been assumed to be slightly compressible in simulations.

It might appear that this list of requirements is too onerous but it will be shown that *the minimum of the axial component of the Eulerian logarithmic strain tensor* satisfies all of these properties and is therefore the standard strain measure advocated here. This minimum value is a compressive strain component and thus will be negative, as shown in figure 1. For convenience, however, the absolute value of the strain measure will be exclusively considered here.

This identification still leaves the issue of the standard being immediately and conveniently available to experimenters in a laboratory setting unresolved, given that the minimum axial component of the Eulerian logarithmic strain tensor must be determined using finite element techniques. It is shown here, however, that an empirical relationship between the minimum of the axial component of the Eulerian logarithmic strain tensor and the probe radius and displacement is an excellent predictor of the strain measure. One essential feature of this relationship is that the linear theory is recovered on restriction to infinitesimal deformations, adopting the axiom that any nonlinear theory should recover its linear form when infinitesimal inputs are being considered. It will be shown here that the minimum axial linear strain *s*_lin_ can be obtained analytically for the problem of spherical indentation and is given by

1.1 $$s_{\mathrm{lin}}=0.452\sqrt{\frac{D}{R}}.$$

This measure will therefore be used as the leading-order term in the proposed empirical formula for indentation strain, *s*_ind_. It will be shown that

1.2 $$s_{\mathrm{ind}}=0.452\sqrt{\frac{D}{R}}+0.102\frac{D}{R}$$

is an excellent fit for the empirical relationship between the strain obtained from finite element simulations of indentation and the displacement of the probe. This formula is therefore proposed as a reliable and efficient method of quantifying strain for indentation experiments on isotropic soft tissue and, if implemented, will enable different indentation experiments to be speedily compared and referenced, something that is not possible currently. Given that indentation methods have made, and will continue to make, significant contributions to the determination of the mechanical response of brain tissue, an unambiguous and easily computed strain standard such as (1.2) is sorely needed.

Another essential feature of the measure advocated here is that it is derived from simulations that have a sound physical basis. All the simulations use models that closely mimic the force–displacement data obtained using a bespoke, accurately calibrated micro-indentation device from fresh murine brains that were carefully harvested. Full details of the experimental protocols used are given in §5.

## 2. Strain measures

A deformation is a smooth, orientation-preserving mapping **x**=*χ*(**X**) from a reference configuration in which a particle has position **X** to a deformed configuration in which the particle has position **x**. Strain can be defined as a measure of the size of the deformation from the reference to the current configurations, with a necessary condition on any proposed tensorial strain measure being that it recovers the infinitesimal strain tensor *ϵ* on restriction to infinitesimal deformations. It therefore seems natural to define strain in terms of the deformation gradient tensor **F**≡∂**x**/∂**X**, which admits the polar decomposition **F**=**RU**=**VR** where the rotation tensor **R** is a proper orthogonal tensor and the stretch tensors **U**,**V** are positive definite and symmetric. The tensors **U**,**V** have the same positive eigenvalues λ_*i*_, *i*=1,2,3, which are the principal stretches. The so-called Cauchy–Green strain tensors, **B** and **C**, are defined as follows:

$$\text{B}={\text{FF}}^{T}={\text{V}}^{2}\;\mathrm{and}\;\text{C}={\text{F}}^{T}\text{F}={\text{U}}^{2},$$

and their invariants have the form

2.1 $$I_{1}=\lambda_{1}^{2}+\lambda_{2}^{2}+\lambda_{3}^{2},\;I_{2}=\lambda_{1}^{2}\lambda_{2}^{2}+\lambda_{1}^{2}\lambda_{3}^{2}+\lambda_{2}^{2}\lambda_{3}^{2}\;\mathrm{and}\;I_{3}=\lambda_{1}^{2}\lambda_{2}^{2}\lambda_{3}^{2}.$$

For the incompressible materials of interest here, *I*_3_≡1.

Other strain measures **E** that have been proposed have measure 0 in the reference configuration. The more popular are essentially polynomial functions of either **U**, the so-called Lagrangian strain tensors

2.2 $$\text{E}=\left\{\begin{array}{cc} \frac{1}{2}({\text{U}}^{n}-1) & \text{if }n\neq 0\\ \ln\;\text{U} & \text{if }n=0 \end{array}\right\}$$

and the corresponding Eulerian strains, polynomial functions of **V**,

2.3 $$\text{E}=\left\{\begin{array}{cc} \frac{1}{2}({\text{V}}^{n}-1) & \text{if }n\neq 0\\ \ln\;\text{V} & \text{if }n=0 \end{array}\right\},$$

where *n* is a real number. See, for example, Neff *et al.* [10] and Holzapfel [11] for further insight and motivation for these forms.

The documentation supporting commercial finite element codes are not as forthcoming and clear on this crucial concept, with definitions and motivation for different choices of strain split between the Theory Guide and the User’s Guide and between different sections of these two guides in the documentation for Abaqus 6.14, for example. Without motivation, §4.2.1 of the User’s Guide states that, for geometrically nonlinear analysis, the Eulerian logarithmic strain (2.3)_2_ defined as

2.4 $$\ln\text{V}=\sum_{i=1}^{3}\ln\;\lambda_{i}v_{i}⊗v_{i},$$

where ***v***_*i*_ are the principal stretch directions in the current configuration, is the default strain measure, and this default measure will be used for convenience here also. This strain measure is sometimes associated with Hencky [12], who proposed that certain nonlinearly elastic materials be modelled using a strain-energy function of the same form as the classical, linear strain-energy function for homogeneous isotropic materials but with the principal infinitesimal strains replaced by the principal values of (2.4). This strain-energy function has received much attention in the literature (e.g. [13–16] and references therein).

Strain is uniquely defined for infinitesimal deformations. Let ***u***=***x***−***X*** denote the displacement of a typical particle from the reference configuration to the current. Let ***H***≡∂***u***/∂***X***. Therefore,

2.5 F=I+H.

Let $\epsilon \equiv (\frac{1}{2})(H+H^{T})$, and in the linear theory it is assumed that

2.6 ϵ=∥H∥≪1,

where ∥⋅∥ denotes the *sup* norm. A *sine qua non* of any nonlinear theory is that the linear theory be recovered on restriction to infinitesimal inputs and it is easily shown that all of the strain measures (2.2), (2.3) reduce to ***ϵ*** for linear deformations. This essential property is incorporated into the strain measure for nonlinear indentation proposed here and is what sets this new measure apart from those previously proposed.

The focus here is on strain measures for *spherical* indentation, the method of choice for very soft tissue such as brain tissue because there are no sharp edges that could cause damage. There is a natural length-scale associated with spherical probes that is easy to measure: their radius *R*. A less obvious length-scale is the radius of the contact region between the probe and specimen *a*, which is much more difficult, if not impossible, to measure and which, in general, depends on the material being tested. Despite the complications associated with the determination of *a*, the ratio of the two radii *a*/*R* seems a natural measure of strain for spherical indentation and has been advocated as such by Iwashita *et al.* [17] and Chyasnavichyus *et al.* [18], for example. Alternatively, Lin *et al.* [19] proposed that 0.2 *a*/*R* is a suitable measure for soft tissue modelling, an empirical strain measure originally proposed by Tabor [20] for the spherical indentation of perfectly plastic materials.

These strain measures are not easily usable in a laboratory setting as the radius of contact *a* is not a parameter that is easily determined. It makes more sense to define a strain measure in terms of the probe displacement *D* and probe radius *R*. However, it is not clear what function of the ratio *D*/*R* is the most appropriate. Guidance in this regard is given from a consideration of the linear theory discussed next.

## 3. Indentation and the linear theory

Brain tissue is assumed here to be incompressible, due to its high water content; isotropic, for analytical purposes; and elastic, using insights gained from experimentation. Before considering general indentation experiments, it seems sensible that the linear regime be first clearly identified.

Consider then a half-space of a linearly elastic, isotropic material, with a shear modulus *μ* and a Poisson’s ratio *η* indented normally by an axisymmetric probe. Following Sneddon [21], centre a cylindrical coordinate system with coordinates (*ρ*, *θ*, *z*) at the indenter tip, with the half-space corresponding to *z*≥0. Let *a* denote the radius of contact of the indenter, whose profile is defined by *z*=*f*(*ρ*/*a*), and *D* the depth to which the half-space is penetrated by the indenter. Subject to the following boundary conditions on the half-space surface:

3.1 $$\begin{array}{rlr} \sigma_{\rho z}(\rho ,0) & =0,\;\sigma_{\theta z}(\rho ,0)=0,\;u_{z}=D-f\left(\frac{\rho }{a}\right), & \;0\leq \rho \leq a\\ \;\text{and}\;\sigma_{\rho z}(\rho ,0) & =\sigma_{\theta z}(\rho ,0)=\sigma_{zz}(\rho ,0)=0, & \;\rho >a, \end{array}\}$$

using an obvious notation for cylindrical components of the stress and the displacement, Sneddon [21] obtained the following displacement field as the solution to the equations of equilibrium:

3.2 $$\begin{array}{rl} u_{\rho }(\rho ,z) & =-\frac{a}{2(1-\eta )}\int_{0}^{\infty }J_{1}(\xi \rho )(1-2\eta -\xi z)\psi (\xi a)\;\exp^{-\xi z}\;d\xi ,\\ u_{\theta } & =0\\ \;\text{and}\;u_{z}(\rho ,z) & =\frac{a}{2(1-\eta )}\int_{0}^{\infty }J_{0}(\xi \rho )[2(1-\eta )+\xi z]\psi (\xi a)\;\exp^{-\xi z}\;d\xi , \end{array}\}$$

where *J*_0_,*J*_1_ denote Bessel functions of the first kind of order 0 and 1, respectively, and

3.3 $$\psi (s)\equiv \int_{0}^{1}\chi (t)\cos(st)\;dt,\;\chi (t)\equiv \frac{2D}{\pi }-\frac{2t}{\pi }\int_{0}^{t}\frac{f^{\prime }(x)\;dx}{\sqrt{t^{2}-x^{2}}}.$$

The function *ψ* will be called the displacement potential here and *χ*, the shape function. To ensure that the axial normal stress is finite within the vicinity of the radius of contact, Sneddon [21] imposed the restriction that

3.4 χ(1)=0,

which will also be assumed here. It will also be assumed that *χ*^′^(*t*)≤0 and therefore

3.5 $$0\leq \chi (t)\leq \frac{2D}{\pi },\;t\in [0,1].$$

The cylindrical polar coordinates of the corresponding strain field are therefore

3.6 $$\begin{array}{rl} \epsilon_{\rho \rho }(\rho ,z) & =-\frac{a}{2(1-\eta )}\int_{0}^{\infty }\xi J_{0}(\xi \rho )(1-2\eta -\xi z)\psi (\xi a)\;\exp^{-\xi z}\;d\xi \\ & \;+\frac{a}{2(1-\eta )\rho }\int_{0}^{\infty }J_{1}(\xi \rho )(1-2\eta -\xi z)\psi (\xi a)\;\exp^{-\xi z}\;d\xi ,\\ \epsilon_{\theta \theta }(\rho ,z) & =\frac{u_{\rho }(\rho ,z)}{\rho }=-\frac{a}{2(1-\eta )\rho }\int_{0}^{\infty }J_{1}(\xi \rho )(1-2\eta -\xi z)\psi (\xi a)\;\exp^{-\xi z}\;d\xi ,\\ \epsilon_{zz}(\rho ,z) & =\frac{a}{2(1-\eta )}\int_{0}^{\infty }\xi J_{0}(\xi \rho )(2\eta -1-\xi z)\psi (\xi a)\;\exp^{-\xi z}\;d\xi ,\\ \epsilon_{\rho z}(\rho ,z) & =-\frac{az}{1-\eta }\int_{0}^{\infty }\xi^{2}J_{1}(\xi \rho )\psi (\xi a)\;\exp^{-\xi z}\;d\xi \\ \;\text{and}\;\epsilon_{\rho \theta } & =\epsilon_{\theta z}=0, \end{array}\}$$

with therefore

$$\text{tr}\;e=\frac{a(2\eta -1)}{1-\eta }\int_{0}^{\infty }\xi J_{0}(\xi \rho )\psi (\xi a)\;\exp^{-\xi z}\;d\xi .$$

### 3.1. Sneddon’s force–displacement formula

The *surface* strains are given by setting *z*=0 in (3.6) and are given by

3.7 $$\begin{array}{rl} \epsilon_{zz}(\rho ,0) & =\frac{a(2\eta -1)}{2(1-\eta )}\int_{0}^{\infty }\xi J_{0}(\xi \rho )\psi (\xi a)\;d\xi ,\\ \epsilon_{\theta \theta }(\rho ,0) & =\frac{u_{\rho }(\rho ,0)}{\rho }=\frac{a(2\eta -1)}{2\rho (1-\eta )}\int_{0}^{\infty }J_{1}(\xi \rho )\psi (\xi a)\;d\xi \\ \;\text{and}\;\epsilon_{\rho \rho }(\rho ,0) & =\frac{a(2\eta -1)}{2(1-\eta )}\left(\int_{0}^{\infty }\xi J_{0}(\xi \rho )\psi (\xi a)\;d\xi -\frac{1}{\rho }\int_{0}^{\infty }J_{1}(\xi \rho )\psi (\xi a)\;d\xi \right). \end{array}\}$$

It follows that

3.8 $$\text{tr}\;e(\rho ,0)=2\epsilon_{zz}(\rho ,0),$$

and therefore the surface axial normal stress has the form

3.9 $$\sigma_{zz}(\rho ,0)=\frac{2\mu }{1-2\eta }\epsilon_{zz}(\rho ,0)=\frac{a\mu }{\eta -1}\int_{0}^{\infty }\xi J_{0}(\xi \rho )\psi (\xi a)\;d\xi .$$

Integrating both sides over the contact area yields Sneddon’s force–displacement relation

3.10 $$P=\frac{2\mu }{1-\eta }\pi a\int_{0}^{1}\chi (t)\;dt,$$

where *P* is the force exerted by the indenter.

### 3.2. Incompressible materials

The practical difficulties in determining Poisson’s ratio for soft tissue mean that it is rarely accurately known, with perfect incompressibility commonly assumed to circumvent this lack of knowledge. Letting $\eta \to \frac{1}{2}$ therefore yields

3.11 $$\begin{array}{rl} \epsilon_{\rho \rho }(\rho ,z) & =az\int_{0}^{\infty }\xi^{2}J_{0}(\xi \rho )\psi (\xi a)\;\exp^{-\xi z}\;d\xi -\frac{az}{\rho }\int_{0}^{\infty }\xi J_{1}(\xi \rho )\psi (\xi a)\;\exp^{-\xi z}\;d\xi ,\\ \epsilon_{\theta \theta }(\rho ,z) & =\frac{az}{\rho }\int_{0}^{\infty }\xi J_{1}(\xi \rho )\psi (\xi a)\;\exp^{-\xi z}\;d\xi ,\\ \epsilon_{zz}(\rho ,z) & =-az\int_{0}^{\infty }\xi^{2}J_{0}(\xi \rho )\psi (\xi a)\;\exp^{-\xi z}\;d\xi ,\\ \epsilon_{\rho z}(\rho ,z) & =-2az\int_{0}^{\infty }\xi^{2}J_{1}(\xi \rho )\psi (\xi a)\;\exp^{-\xi z}\;d\xi \\ \;\text{and}\;\epsilon_{\rho \theta } & =\epsilon_{\theta z}=0, \end{array}\}$$

with now tr ***e***=0. The force–displacement relation (3.10) becomes

3.12 $$P=4\mu \pi a\int_{0}^{1}\chi (t)\;dt,$$

Physical intuition and finite element simulations suggest that the largest strain in indentation occurs along the ray beneath the centre of the probe. If *ρ*=0, and noting that $\lim_{\rho \to 0}(J_{1}(\xi \rho )/\rho )=\xi /2$, the normal strain field beneath the probe is therefore given by

3.13 $$\begin{array}{rl} \epsilon_{\rho \rho }(0,z) & =\epsilon_{\theta \theta }(0,z)=az^{2}\int_{0}^{1}\chi (t)\frac{z^{2}-3a^{2}t^{2}}{{\left(z^{2}+a^{2}t^{2}\right)}^{3}}\;dt\\ \;\text{and}\;\epsilon_{zz}(0,z) & =-2az^{2}\int_{0}^{1}\chi (t)\frac{z^{2}-3a^{2}t^{2}}{{\left(z^{2}+a^{2}t^{2}\right)}^{3}}\;dt, \end{array}\}$$

using the identity

$$\int_{0}^{\infty }\xi^{2}\;\exp^{-\xi z}\cos(\xi at)\;d\xi =\frac{2z(z^{2}-3a^{2}t^{2})}{{\left(z^{2}+a^{2}t^{2}\right)}^{3}},$$

from Bateman’s Table of Transforms [22]. The shear strains are identically zero beneath the centre of the indenter and therefore the normal strains (3.13) are principal strains. Note that the axial strain component is twice as large as both the radial and azimuthal components.

### 3.3. Spherical probes

Spherical indentation is the procedure of choice in many indentation experiments and is therefore the only probe profile considered here. Sneddon [21] showed that, for spherical probes of radius *R* (>*a*), the shape function has the following form:

3.14 $$\chi (t)=\frac{2D}{\pi }-\frac{at}{\pi }\log\frac{R+at}{R-at}\;\mathrm{and}\;D=\frac{a}{2}\log\frac{R+a}{R-a}.$$

However, evaluation of the integral in (3.13) to determine the axial strain component is extremely tedious and will not be attempted here. An alternative strategy is adopted instead.

Assume that

3.15 $$\epsilon \equiv \frac{a}{R}\ll 1.$$

Expanding the righ-hand side of (3.14)_2_ as a perturbation series in *ϵ* and truncation after the leading-order term yields

3.16 $$\frac{a}{R}=\sqrt{\frac{D}{R}},$$

which is exactly that assumed in the Hertzian theory of contact [23]. It follows that the logarithmic function in (3.14)_1_ can therefore be approximated as

3.17 $$\log\frac{R+at}{R-at}=2\epsilon t,$$

with the shape function (3.14) now having the form

$$\chi (t)=\frac{2D}{\pi }(1-t^{2})$$

and therefore very large spherical probes behave as if they were paraboloids of revolution [24].

First note that the force–displacement relation (3.12) can now be written in the form

3.18 $$P=\frac{16}{3}\mu R^{1/2}D^{3/2},$$

which is the Hertzian force–displacement relation for incompressible materials.

Evaluation of the axial strain component (3.13) assuming Hertzian contact is now straightforward with

$$\epsilon_{zz}(0,z)=\frac{4D}{\pi a}\left(\frac{{\bar{z}}^{2}}{1+{\bar{z}}^{2}}-\bar{z}\tan^{-1}\frac{1}{\bar{z}}\right),$$

using the notation $\bar{z}\equiv z/a$. The qualitative features of this strain are easily obtained: following a zero initial compressive strain, there is a minimum compressive strain of value −1.421(*D*/*πa*) occurring at *z*=0.548*a*. Thus the minimum compressive strain for spherical indentation also occurs at *z*=0.548*a*, with an absolute maximum value

3.19 $$s_{\mathrm{lin}}=0.452\frac{D}{a}=0.452\frac{a}{R}=0.452\sqrt{\frac{D}{R}}.$$

Thus the linear theory provides the appropriate function of the intuitive strain measure *D*/*R* that should be used as the basis of any nonlinear strain measure for indentation. It would appear that the natural strain measure for nonlinear indentation should be of the form

3.20 $$s_{\mathrm{ind}}=0.452\sqrt{\frac{D}{R}}+c\frac{D}{R},\;\text{constant}\;c,$$

expanding in a Maclaurin series in $\sqrt{D/R}$ and truncating after the second-order term. It will be shown empirically that *c* is substantially invariant under the choice of indenter radius *R* and the choice of material model.

## 4. Models of nonlinear elasticity

It follows from (3.19) that the linear regime for indentation experiments on elastic, incompressible, isotropic half-spaces is characterized by the condition

4.1 $$s_{\mathrm{lin}}=0.452\sqrt{\frac{a}{R}}\ll 1,$$

which is the specification for indentation of the general definition of linearity given in (2.6). Note that assumption (3.15), which leads to the Hertzian contact relation (3.16), is consistent with linearity. From a practical point of view, this linearity assumption effectively means that the displacement of the probe must be at least a thousand times smaller that the probe radius. This can be seen by substituting $0.452\sqrt{a/R}=\frac{1}{100}$, a value that is in the upper range of geometries consistent with the linearity restriction (4.1), into (3.14)_2_ to obtain *D*/*R*=0.0005. So the probe displacement must be of a different scale from that of the probe radius before the linear theory can be applied. Thus, for example, only nano-indentation in displacement is consistent with linearity for micro-indentation in terms of the probe radius.

This mismatch of scalings for indentation experiments effectively means that all soft tissue behaves as a nonlinearly elastic material, the mechanical response of which is completely determined by specification of the strain-energy function *W* per unit of undeformed volume. As the material is assumed incompressible and isotropic, the strain energy can be written as an arbitrary function of any of the two independent invariants of the strain tensors discussed in §2. The invariants of the Cauchy–Green strain tensors *I*_1_,*I*_2_ defined in (2.1) are a classical choice with the following two specifications widely used, where *c*_10_,*c*_01_ are material constants to be determined from experiment:

1. the neo-Hookean model with *W*=*c*_10_(*I*_1_−3);
2. the Mooney–Rivlin model with *W*=*c*_10_(*I*_1_−3)+*c*_01_(*I*_2_−3).

Ogden [25] has advocated using the invariants of the strain tensors (2.2), (2.3) with a typical strain energy of the form

4.2 $$W=\sum_{i=1}^{n}c_{i}(\lambda_{1}^{\alpha_{i}}+\lambda_{2}^{\alpha_{i}}+\lambda_{3}^{\alpha_{i}}-3),$$

where *c*_*i*_ are constants to be determined from experiment.

The numerical difficulties associated with locally enforcing the incompressibility constraint in the displacement formulation of the finite element method means that a slightly compressible version of the perfectly incompressible neo-Hookean, Mooney–Rivlin and Ogden models is often assumed when simulating soft tissue. In Abaqus, for example, the slightly compressible form of the Mooney–Rivlin strain energy is implemented as

4.3 $$W=c_{1}(I_{1}^{*}-3)+c_{2}(I_{2}^{*}-3)+\frac{1}{D_{1}}{\left(I_{3}^{1/2}-1\right)}^{2},$$

where $I_{1}^{*}\equiv I_{3}^{-1/3}I_{1},\;I_{2}^{*}\equiv I_{3}^{-2/3}I_{2}$. The parameter *D*_1_ is usually called the compressibility factor and is related to the infinitesimal bulk modulus *κ* through the relation *D*_1_≡2/*κ*. It is assumed here that *κ*=10 000×*μ* for brain tissue (equivalent Poisson’s *ratio*=0.49995), where *μ* is the infinitesimal shear modulus. The Abaqus slightly compressible formulation of the incompressible Ogden material (4.2) has the form

$$W=\sum_{i=1}^{n}\frac{2\mu_{i}}{\alpha_{i}^{2}}({\left(\lambda_{1}^{*}\right)}^{\alpha_{i}}+{\left(\lambda_{2}^{*}\right)}^{\alpha_{i}}+{\left(\lambda_{3}^{*}\right)}^{\alpha_{i}}-3)+\sum_{i=1}^{n}\frac{1}{D_{i}}{\left(I_{3}^{1/2}-1\right)}^{2i},$$

where $\lambda_{i}^{*}\equiv I_{3}^{-1/6}\lambda_{i}$ and *N*,*μ*_*i*_,*α*_*i*_,*D*_*i*_ are material constants. It was found that only a one-term model of the form

$$W=\frac{2\mu }{\alpha^{2}}({\left(\lambda_{1}^{*}\right)}^{\alpha }+{\left(\lambda_{2}^{*}\right)}^{\alpha }+{\left(\lambda_{3}^{*}\right)}^{\alpha }-3)+\sum_{i=1}^{n}\frac{1}{D_{i}}{\left(I_{3}^{1/2}-1\right)}^{2i},$$

was sufficient to accurately model the indentation experiments considered here.

Experimental data to motivate the analysis are obtained from indentation experiments on murine brain tissue to ensure that the standards proposed here have a sound physical basis.

## 5. Material and methods of experimentation

### 5.1. Experimental apparatus

**Figure 2.**
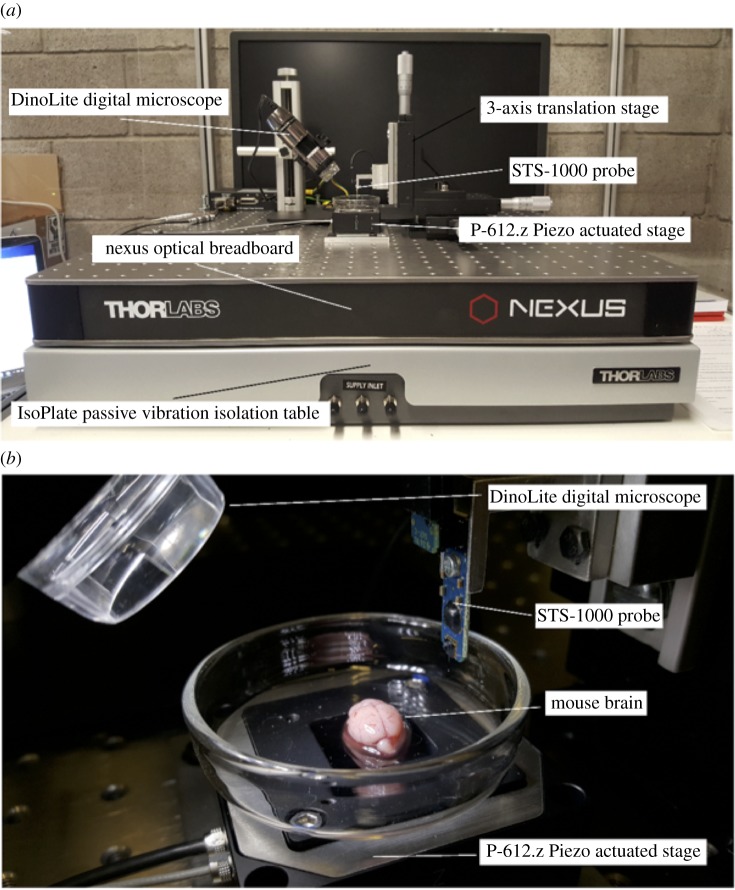
(*a*) Custom-built micro-indentation apparatus detailing the main components outlined in §5.1, and (*b*) a close-up view of the piezo-actuated stage and STS-1000 probe before commencing indentation experiments on mouse brain.

A custom-built micro-indentation device was developed to investigate the local mechanical properties of brain tissue (figure 2). The custom-built device uses a Physik Instrumente P-612.Z piezo actuator stage to translate samples in the vertical direction (100 μm travel range) to impact the FemtoTools STS-1000 force-sensing probe. The piezo actuator uses closed-loop strain gauge control positioning with a closed-loop resolution of 1.5 nm, linearity error 0.2% and repeatability of ±4 nm, calibrated in-house by Physik Instrumente using a Millitron Precision Gauge (calibration certificate supplied with order). The STS-1000 probes have a spherical ruby tip with a 125 μm radius, a resolution of 0.05 μN at 10 Hz. The probes are connected via a proprietary cable to the FT-SC01 Force Acquisition System with a sampling rate of 10 kHz. The FTS-1000 probes are calibrated individually in-house by FemtoTools (calibration certificate supplied with order) and are provided with unique sensor gains (approx. 500 μN V^−1^). The STS-1000 probes are fixed to a 3-axis translation stage with standard micrometers (engraved every 10 μm) for manual positioning of the probe and determination of the contact point. A DinoLite AM7915MZTL (AnMo Electronics Corp., Taiwan) digital microscope is used to image the location of the indentation and to ensure that the indentation area is void of vasculature. All the components mentioned above are fixed to a Nexus optical breadboard which is placed on an IsoPlate passive vibration isolation table (ThorLabs Inc., Newton, NJ, USA). The entire system is placed inside an aluminium-framed plexiglass enclosure (Machine Building Systems Ltd, Co. Westmeath, Ireland).

### 5.2. Tissue preparation

Mice were euthanized by *CO*_2_ inhalation and collected on the day of testing from University College Dublin’s Biomedical Facility. The specimens consisted of six-week-old mixed male and female mice. Mixed-sex mice were used as it has been previously reported that gender has no effect on the compressive response of brain tissue [26]. To perform the indentation experiments, the brains were removed from the animals by making a midline incision through the skin across the top of the head to gain access to the skull. A second midline incision, moving anteriorly from the occipital condyle, was made through the skull using a scalpel. Two lateral incisions were then made at an anterior and posterior point of the midline incision so that the bone could be removed to allow access to the brain. The brain was then separated from the spinal cord and removed from the skull. Following their removal from the skull, the brains were kept hydrated with phosphate buffer saline (PBS), while the surfaces of the cortex and cerebellum were indented. Each brain was continuously hydrated throughout the experiment with PBS. All tests were completed within 6 hours post-mortem in order to reduce the amount of proteolysis and necrosis that has been previously shown to reduce the stiffness of the tissue [27,28].

### 5.3. Indentation protocols

Indentation tests using probes with a spherical ruby tip of radius 125 μm were performed *in vitro* on cerebral cortex (*n*=28) and cerebellum (*n*=30) tissues from six-week-old mice (*n*=6) (figure 2*b*) to a depth of 57.7 μm at a constant velocity of 0.165 mm s^−1^. At this velocity, the STS-1000 probes have a force resolution of 0.0267 μN, providing an overall maximum theoretical error of 0.1% for the force readings. To achieve constant velocity throughout the indentation, first the contact point was established by manually adjusting the probe’s position using the micrometers on the translation stage to bring the indenter tip into contact with the tissue. The position of contact is noted and the indenter tip is retracted 20 μm from this position to allow the piezo actuator to achieve a constant velocity before impacting the sample into the indenter tip. The total indentation depth is equal to 67.7 μm allowing 10 μm for deceleration of the stage, with the final 10 μm of the indentation data therefore discarded due to inertial effects in the force readings. If a force greater than 5 μN was recorded during establishment of the contact point, the indenter was moved to a new location to perform the force measurement. No other preconditioning is performed on the sample as the sample is not preconditioned *in vivo*. All tests were conducted at room temperature (≈22°C) and specimens were discarded after each set of indentations. Two distinct regions, cortex and cerebellum, were chosen in order to show that the strain measure proposed here for indentation experiments are not particular to a specific region of the brain.

### 5.4. Results

**Figure 3.**
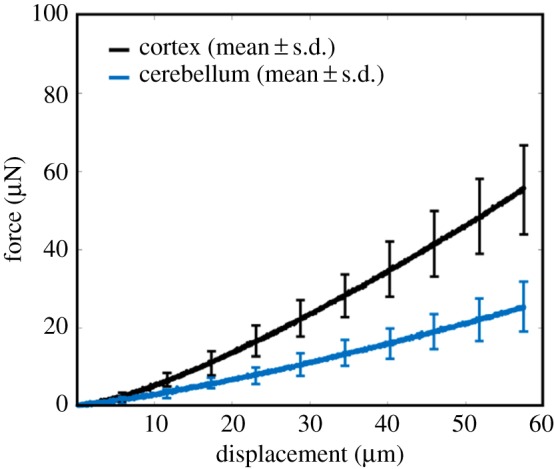
Mean± standard deviation (s.d.) force–displacement curves of the cortex (black) and cerebellum (blue) indentation experiments.

A graphical summary of the results is given in figure 3 and these data will form an essential component of the analysis that follows. No filtering is applied to the data prior to fitting of the mathematical models.

## 6. Determination of constitutive models

An essential feature of any universal measure of strain for indentation experiments is that it should be independent of the specific material model assumed for the brain tissue, given that brain tissue is assumed here to be flat, smooth, homogeneous, isotropic, incompressible and nonlinearly elastic. The three models of such behaviour introduced in §4 were fitted to the experimental data of the last section using inverse finite element techniques described next to determine the optimal values for the constitutive properties of the cerebral cortex and the cerebellum.

**Figure 4.**
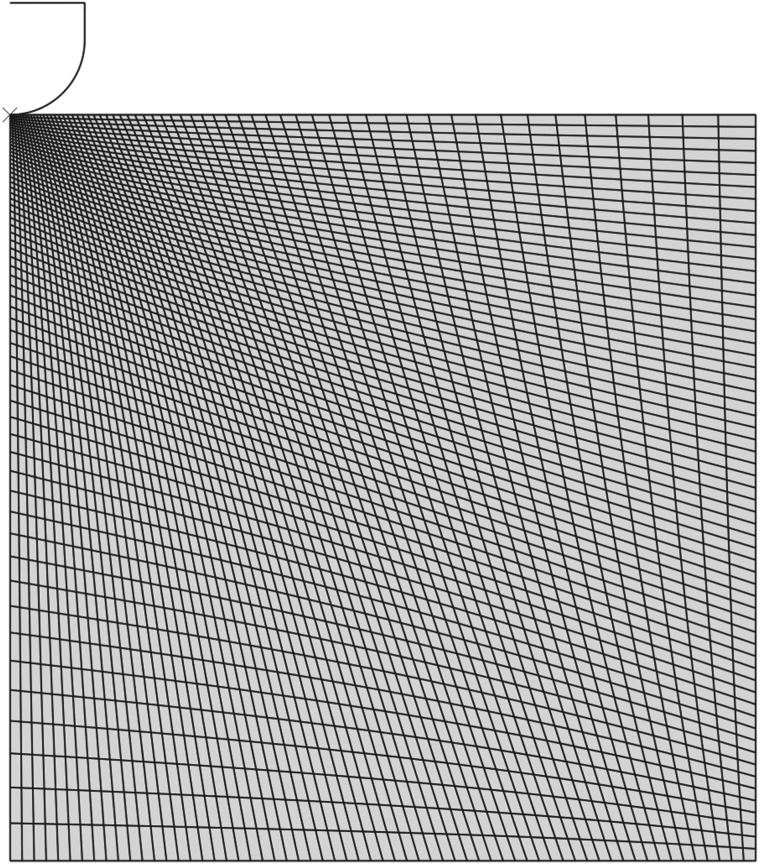
The axisymmetric finite element model in the reference configuration.

Taking advantage of the indenter’s symmetry, axisymmetric analysis was performed using the commercial finite element software Abaqus (Dassault Systemes, RI, USA). The model (figure 4) consisted of a rigid indenter tip geometry, modelled as a 125 μm radius hemisphere consisting of 74 linear RAX2 line elements. The rigid indenter tip was initially in contact with a deformable cylinder, with a radius and height of 1250 μm, consisting of 3844 linear quadrilateral CAX4RH reduced integration, hybrid elements, which represents a sample of brain tissue. A fixed boundary condition was applied to the bottom face of the cylinder, and a displacement boundary condition of 57.7 μm was applied to the indenter tip. The contact area is assumed to be locally perpendicular to the direction of the applied force due to the absence of gyri and sulci in the mouse brain. Nonlinear geometry was used in this analysis due to the large deformation near the contact area. A Matlab script was created to simulate the experiment for a range of shear moduli values between 0.1 and 10 kPa in 0.1 kPa steps for the neo-Hookean model; the Mooney–Rivlin model parameters *c*_01_ and *c*_10_ ranged from 0.1 to 5.1 kPa in 0.6 kPa steps, while the Ogden model shear modulus *μ* ranged from 0.1 to 9.6 kPa in 0.6 kPa steps, and *α* ranged from −13 to 13.6 in increments of 1.4. Poisson’s ratio *η* remained fixed at 0.49995 for all simulations. The material parameters and corresponding force–displacement curves were then stored in separate arrays. These data were then used in the response surface method by employing a custom Matlab function which fits the experimental force–displacement curves to the numerical curves generated for the neo-Hookean, Mooney–Rivlin and Ogden constitutive models, interpolating between the precomputed curves if required. When the error between the two curves reaches the minimum, using the sum of absolute differences method, the program terminates. The best-fit constitutive parameters for the three nonlinear models are given in table 1.

**Table 1.** Model parameters (mean ± s.d.) obtained using inverse finite element techniques.

model parameter	cortex	cerebellum
neo-Hookean		
*c*_10_ (kPa)	1.18±0.23	0.56±0.13
Mooney–Rivlin		
*c*_10_ (kPa)	0.55±0.03	0.27±0.01
*c*_01_ (kPa)	0.58±0.19	0.26±0.11
Ogden		
*μ* (kPa)	2.31±0.43	1.08±0.24
*α*	0.9±0.23	0.6±0.37

**Figure 5.**
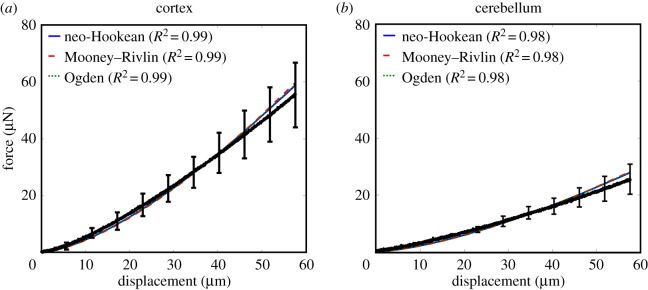
*Mean*±*s*.*d*. force–displacement curves in black with the neo-Hookean (blue), Mooney–Rivlin (dashed red) and Ogden (dotted green) model predictions for (*a*) the cortex and (*b*) the cerebellum.

The excellent fit between these models and the experimental data can be seen in figure 5.

## 7. Invariance of minimum strain

The Eulerian logarithmic strain ln⁡V is the default strain measure in Abaqus. It is proposed here that the minimum axial component of the logarithmic strain tensor (MALS) be used as the standard measure of strain for indentation experiments, thus enabling different sets of indentation data to be compared, contrasted and archived, as needed. The minimum value has the inherent advantage of having a norm structure, in contrast to, say, any average measures of strain that might be alternatively composed.

However, from a practical point of view, the minimum axial logarithmic strain (MALS) as a strain measure would not be practical if it needed to be recalculated for each experiment. Our multifarious simulations have shown that, fortuitously, the MALS is essentially invariant for indentation experiments. Specifically, it would appear that the MALS is invariant under:

1. type of material,
2. location in the brain,
3. variation in material parameters,
4. probe radius, and
5. sample compressibility.

**Figure 6.**
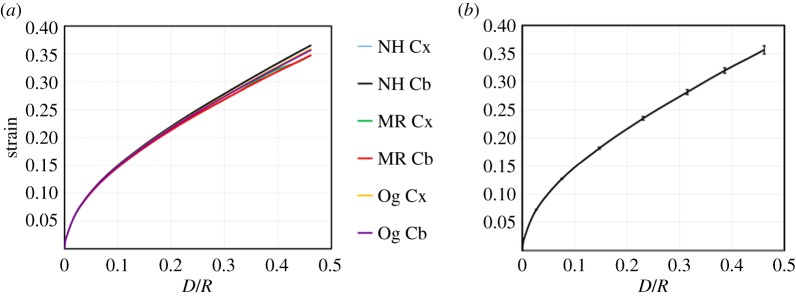
(*a*) Maximum axial logarithmic strain versus *D*/*R* plots showing the invariance of the axial logarithmic strain in finite element simulations of using experimentally derived material properties for brain tissue and the neo-Hookean (NH), Mooney–Rivlin (MR) and Ogden (Og) models for both the cortex (Cx) and cerebellum (Cb). (*b*) The mean ± standard deviation force–displacement curve.

In our simulations, each factor was varied while keeping all the other factors constant at physically realistic values. A sample of these simulations is now presented. Invariance of the MALS with respect to change in the assumed model and location in the brain is evident in figure 6, where the MALS as a function of *D*/*R* is plotted for the neo-Hookean, Mooney–Rivlin and Ogden constitutive models, with parameters given in table 1, with *R*=100 μm and *η*=0.452.

**Figure 7.**
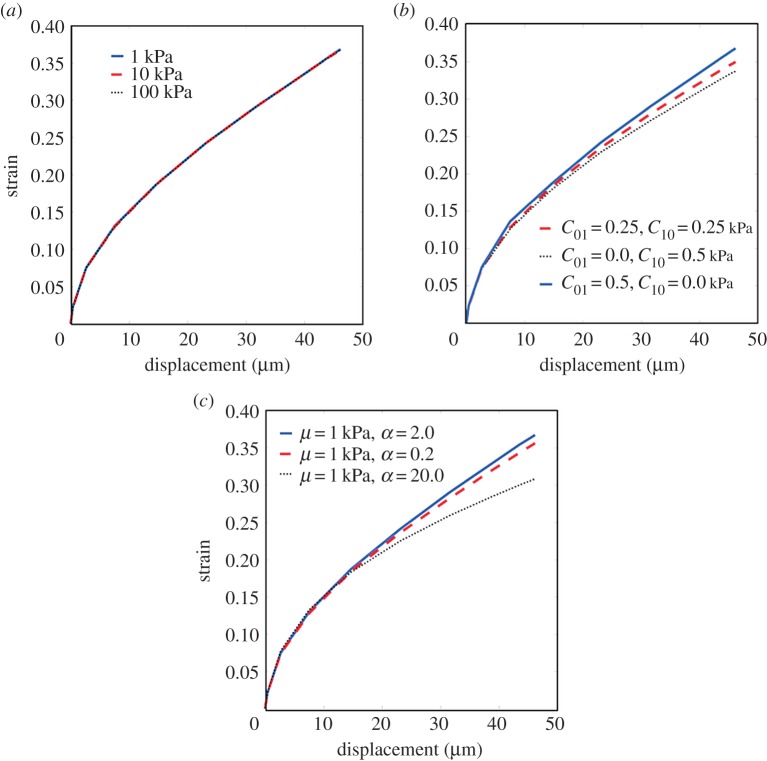
Minimum axial logarithmic strain anddisplacement graphs showing the invariance of the minimum axial logarithmic strain under variation in model parameters for (*a*) the neo-Hookean, (*b*) the Mooney–Rivlin and (*c*) the Ogden models.

In figure 7, the essential invariance of the MALS under variation in values of the material parameters is clear when it is plotted against indentation depth for the range of experimental probe displacements for a number of different instances of the neo-Hookean, Mooney–Rivlin and Ogden models, with again *R*=100 μm and *η*=0.452.

**Figure 8.**
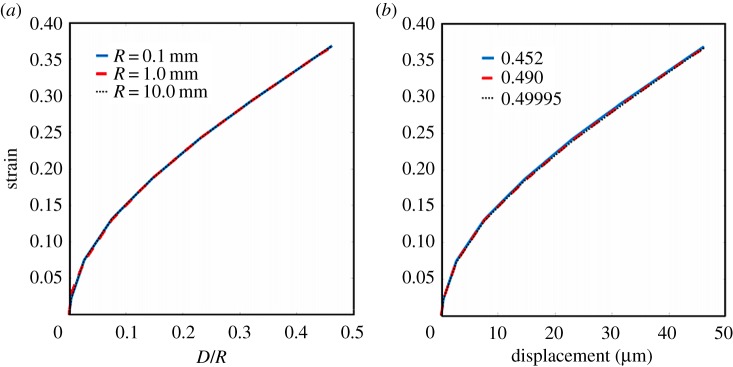
The invariance of minimum axial logarithmic strain–displacement plots under variations of probe radius and compressibility factor.

The invariance under variations of probe radius and compressibility factor is illustrated in figure 8*a*,*b*, respectively. In each case, the material was assumed to be neo-Hookean with a shear modulus of 1 kPa.

## 8. An empirical strain measure

The invariance of the MALS for indentation experiments makes it an ideal candidate for a standard measure of strain for these experiments. Its usefulness would be greatly enhanced if one did not have to use finite element simulations in order to quantify this strain for a given experiment. The simulations performed here will therefore be used as the basis of an empirical relation between the MALS and the displacement and radius of the probe, *D*, *R*. To fix ideas, sample values from the plot of MALS against *D*/*R* displayed in figure 6*b* are given in table 2.

**Table 2.** Sample values of the plot of strain against *D*/*R* given in figure 6*b*.

*D*/*R*	average strain
0.0000	0
0.0040	0.0237
0.0267	0.0732
0.0753	0.1280
0.1466	0.1825
0.2308	0.2356
0.3150	0.2823
0.3863	0.3198
0.4349	0.3441
0.4577	0.3555
0.4616	0.3574

**Figure 9.**
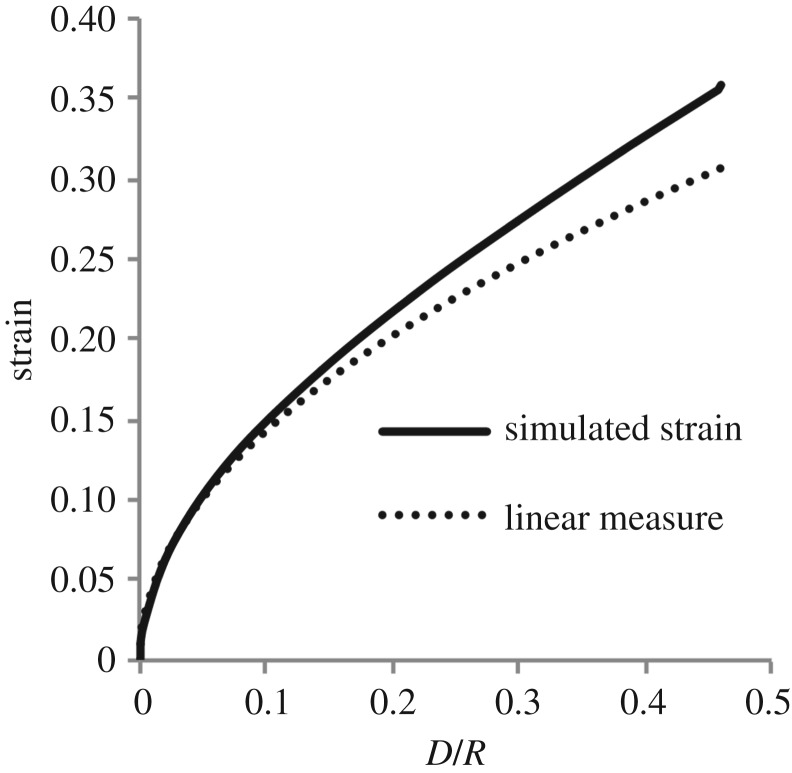
A comparison of the linear measure of axial strain and the simulated nonlinear strains. It is seen that the linear measure is initially an excellent fit with the nonlinear strains but that for larger strains there is a noticeable under-prediction of the nonlinear strain.

The linear theory of §3 will be used to motivate the appropriate form of the proposed empirical relation. Specifically, it will be required that minimum axial compressive strain of the linear theory (3.19) be recoverable from the empirical relation on restriction to infinitesimal strains, obeying the axiom that every nonlinear theory should reduce to the linear on restriction to infinitesimal inputs. To investigate if, coincidentally, the linear strain measure is a good predictor of the nonlinear strains tabulated in table 2, plots of the linear strain

$$0.452\sqrt{\frac{D}{R}}$$

and the simulated nonlinear average strains are given in figure 9. As might be expected, after an initial excellent agreement, the two curves diverge for larger strains, suggesting that a higher-order theory in the parameter $\sqrt{D/R}$ is required.

**Figure 10.**
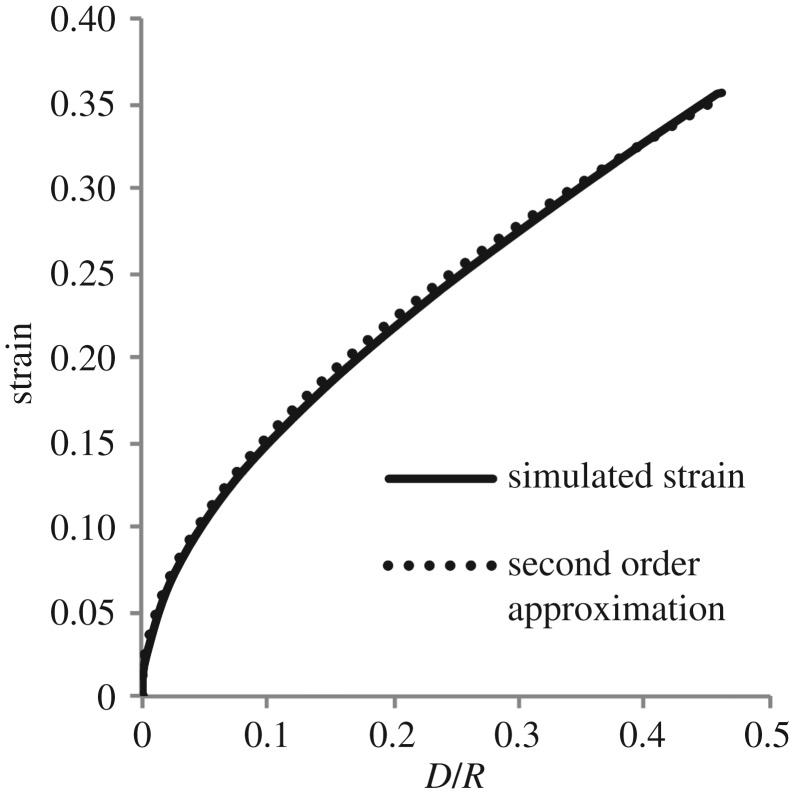
A comparison of the empirical measure of axial strain (8.1) and the simulated nonlinear strains. It is clear that only two terms in the empirical relation are necessary for an excellent predictor of the minimum axial logarithmic strain for the range of strain considered.

It can be seen from figure 10 that the second-order series

8.1 $$s_{\mathrm{ind}}=0.452\sqrt{\frac{D}{R}}+0.102\frac{D}{R},$$

with the coefficient of the second-term determined using regression, is an excellent predictor of the MALS for the range of strain considered. This relation is therefore proposed here as the standard measure of indentation strain for indentation experiments, noting the desirable invariance features of the MALS already discussed and its straightforward expression in terms of the known probe radius *R* and the measured probe displacement *D*. Such a measure is essential in order that different experiments can be easily compared and contrasted, with the need made more urgent by the increasing use of indentation experiments as the primary mode of characterizing the mechanical properties of very soft, biological tissue.

## Supplementary Material

Results Data

## Data accessibility

The supporting data for this manuscript can be found in the Electronic Supplementary Materials file.
