# Correction to ‘An empirical measure of nonlinear strain for soft tissue indentation’

**DOI:** 10.1098/rsos.180703

**Published:** 2018-06-13

**Authors:** D. B. MacManus, M. D. Gilchrist, J. G. Murphy

*R. Soc. open sci.* **4**, 170894. (Published 1 November 2017). (doi:10.1098/rsos.170894)

The incorrect number of mice, their age and number of indentations was incorrectly reported in §5.2 and 5.3 of the original paper. The correct numbers are as follows:

— Indentations were performed on the cerebral cortex (*n *= 13) and cerebellum (*n *= 14) of five week-old mice (*n *= 5).
— The data presented in the electronic supplementary material refers to the wrong figures. This has now been corrected in the attached amended electronic supplementary material.

As the data analysis was actually performed on the numbers presented above, there are no changes to the outcomes/conclusions/results of the original manuscript.

The authors apologize to the journal, reviewers and readers for this oversight.

## Supplementary Material

Corrected ESM

